# Prognostic value of cardiac troponin in dialysis patients with paroxysmal supraventricular tachycardia

**DOI:** 10.1097/MD.0000000000030513

**Published:** 2022-09-09

**Authors:** Chih-Kai Wang, Chieh-Ching Yen, Shou-Yen Chen, Hsiang-Yun Lo, Chip-Jin Ng, Chung-Hsien Chaou

**Affiliations:** a Department of Emergency Medicine, Chang Gung Memorial Hospital, Linkou Branch, Taoyuan, Taiwan; b College of Medicine, Chang Gung University, Taoyuan, Taiwan; c College of Medicine, National Yang-Ming University, Taipei, Taiwan; d Chang Gung Medical Education Research Center, Taoyuan, Taiwan.

**Keywords:** dialysis, emergency department, tachyarrythemia, troponin

## Abstract

A rise in cardiac troponin I (cTnI) is common in supraventricular tachycardia (SVT). While troponin elevation in SVT is thought to be a predictor of future adverse events in patients with prior coronary artery disease, the prognostic significance of cTnI in end-stage kidney disease (ESKD) patients with SVT are not known. We aimed to examine the prognostic significance of cTnI in ESKD patients presenting with SVT in the emergency department. This was a retrospective, multiple-center observational study utilizing regularly collected electronic medical records. We screened electronic medical records of all dialysis patients presenting to the emergency departments in 5 hospitals over 12 years with SVT. These patients were divided into whether cTnI was tested, and were further stratified into the cTnI-positive and cTnI-negative groups. The primary outcome of the study was the 3-year risk of major adverse cardiovascular events (MACE). Sixty-two patients were qualified for inclusion. Fifty-seven patients (91.9%) were tested for cTnI, and 5 patients were not. Patients with the cTnI test were older (*P* = .03) and had a longer length of hospital stay (*P* < .001). Forty-seven patients (82.5%) had a positive result, and 10 (17.5%) had a negative result. A history of hypertension (*P* = .013) and decreased left ventricular ejection fraction (*P* = .048) were the independent predictors of cTnI elevation. After a mean follow-up period of 20.6 ± 14.7 months, there were no differences in 3-year MACE between patients with or without elevated cTnI levels in Kaplan–Meier analysis (*P* = .34). A history of coronary artery disease was the only independent predictor of 3-year MACE (*P* = .017). Through the subgroup analysis, a history of coronary artery disease (HR 2.73; CI 1.01–7.41; *P* = .049) remained an independent risk factor for 3-year MACE in patients with elevated cTnI levels. A large proportion (82.5%) of troponin elevation was observed in ESKD patients with SVT, but it had a poor correlation with MACE.

## 1. Introduction

Supraventricular tachycardia (SVT) is a common arrhythmia seen in the emergency department (ED), with the prevalence and incidence 2.25/1000 and 35/100,000 person-years, respectively.^[1–4]^ It is commonly due to reentry that atrioventricular nodal reentrant tachycardia and atrioventricular reentrant tachycardia comprise major causes.^[5]^ SVT is thought to be rarely life-threatening and mostly has a benign course.^[6]^ Patients with SVT present to the ED typically complained of chest pain, palpitation, dyspnea, dizziness, or syncope.^[7]^ These symptoms are commonly sudden-onset and paroxysmal.^[6]^ These patients are discharged from the ED after conversion to normal sinus rhythm.

During the ED stay of SVT patients, some cases may receive laboratory testing and radiologic examination, including electrolyte, chest radiography, and cardiac enzyme, such as cardiac troponin, especially in patients with cardiovascular diseases. Previous studies have shown that troponin was not a good predictor for coronary artery disease (CAD) in SVT patients.^[8]^ Routine troponin exam in PSVT patients with low risk for coronary artery disease may lead to prolonged length of ED stay, unnecessary exam, and hospitalization.^[8–12]^ However, 1 retrospective study showed elevated troponin I level appear to be associated with adverse outcomes in SVT patients with a history of CAD.^[13]^ This produces doubts as to the prognostic significance of troponin in patients with a huge burden of cardiovascular disease.

Patients with end-stage kidney disease (ESKD) under dialysis are reported to have cardiac disease-related mortality rates of 18.4 per 100 patients-years, and 10- to 30-folds increased risk of cardiovascular death compared with the general population.^[14]^ SVT was present in 41% of these patients,^[15]^ and they frequently receive many investigations in ED owing to a high prevalence of cardiovascular disease. The exact mechanism of arrhythmias in ESKD patients is not clear, but it has been proposed that autonomic dysfunction along with specific cardiac conduction fibrosis contribute to potential reasons.^[16]^ There appears to be an association between arrhythmia and clinical properties such as age, known cardiovascular disease, structural heart disease, hypertension, diabetes mellitus, duration of dialysis, and electrolyte imbalance.^[16]^ Notably, several mechanisms of arrhythmia in ESKD could be attributable to the dialysis procedure itself, in which risk factors consisted of fluctuation in fluid volume and electrolyte level, pro-inflammatory state, and persistent myocardial injury from uremia or other toxins.^[17]^ This theory was backed up by Rantanen et al^[15]^ that tachyarrhythmia was more frequently found during dialysis and the immediate postdialytic period than the rest of the interdialytic period.

While ESKD is a high-risk group for SVT, and cardiac enzymes are often used for risk stratification and diagnosis among patients with various kinds of arrhythmia, there has been little research studying the role of cardiac enzymes in the risk stratification of future adverse outcomes in this population. In the current study, we aimed to examine the prognostic significance of troponin in ESKD patients presenting with SVT in the ED.

## 2. Material and Methods

### 2.1. Study design and setting

This was a retrospective, multiple-center observational study utilizing regularly collected electronic medical records (EMRs). The study site was the ED of 5 hospitals using the same EMR system in Taiwan, including 2 tertiary medical centers and 3 regional hospitals. The study sites’ total capacity was over 9000 beds and an annual ED visit of 500,000 patients. The study period was between January 1, 2009 and May 31, 2021. This study was approved by the Chang Gung Medical Foundation Institutional Review Board (IRB no. 202100707B0) and was qualified for a waiver of informed consent.

### 2.2. Patient selection and data collection

**Figure 1.**
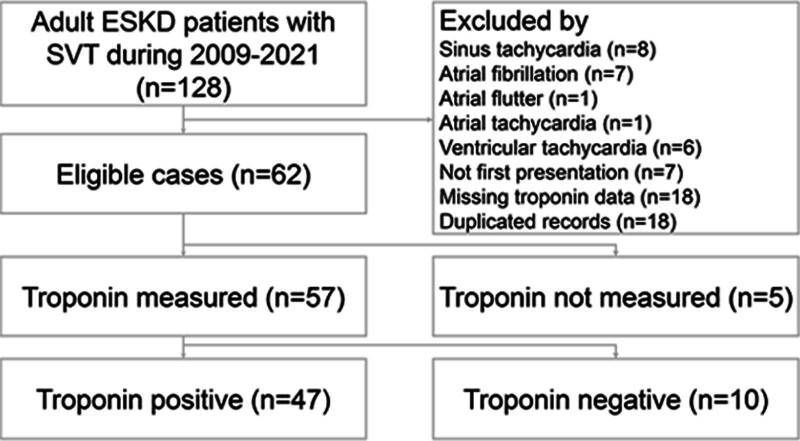
Flow chart of patient selection.

We first identified all patients with International Classification of Diseases (ICD)-9 code 427.0 and ICD-10 code I471 of SVT who presented to the ED through a computerized search from the EMR system. Adult ESKD patients were subsequently identified using codes of 585.6 and N18.6. All EMRs of the above patients were then reviewed by 2 emergency physicians (C-K.W. and C.-C.Y.).The patients whose age younger than 18-year-old, non-dialysis status, duplicate records, incomplete medical records, missing troponin data, not atrioventricular reentry tachycardia or atrioventricular node reentry tachycardia in the electrocardiogram, or not the first presentation of SVT in the ED were excluded (Fig. 1). We collected the relevant variables, including age, gender, vital signs, types of dialysis, symptoms, and signs, laboratory results, electrocardiograms, chest radiographs, length of hospital stay, underlying diseases, medication therapies, whether receiving radiofrequency ablation, and disposition. A peak troponin value was defined as the highest value if patients underwent followed troponin testing. Further cardiac investigations were also collected, including treadmill exercise test, thallium scan, and cardiac catheterization.

### 2.3. Measurable outcomes

The outcome variables included hospital length of stay, percentage of admission, mortality, major adverse cardiac event (MACE) in 6 weeks and 3 years. MACE was defined as ischemic stroke, admission due to acute decompensated heart failure, acute coronary syndrome, revascularization, coronary artery bypass grafting (CABG), and all-cause mortality. The primary outcome of the study was the 3-year risk of MACE. We also evaluated MACE within 6 weeks as a short-term prognosis.

### 2.4. Measurement of cardiac troponin I

The cardiac troponin I (cTnI) level was measured with UniCel DxI 800 immunoassay analyzer (Beckman Coulter DxC880i). The assay has a minimum detectable concentration of <0.01 ng/mL, and the cutoff level (coefficient of variation of ≤10%) for positivity is 0.04 ng/mL.

### 2.5. Statistical analysis

All data were entered into a Microsoft Excel database (Microsoft Inc., Redmond, WA, USA) and analyzed with IBM SPSS Statistics 20 (IBM Corp., Armonk, NY, USA). Patients were divided into 2 groups of whether cTnI was tested. Further, patients with cTnI blood testing were divided into 2 subgroups: cTnI-positive (≥0.04 ng/mL) and cTnI-negative. Categorical data were analyzed using Fisher exact test and numerical data using the Mann–Whitney *U* test. Univariate logistic regression analysis was performed to assess the risk factor for elevated cTnI levels. Multivariate logistic regression was performed with stepwise selection to identify independent variables, with a *P* value cutoff of 0.1. Univariate Cox regression analysis was employed to identify independent risk factors for MACE in 3 years. Multivariate Cox regression analysis was not performed because only 1 variable of a history of coronary artery disease was selected using stepwise selection with a *P* value cutoff of 0.1. Kaplan–Meier survival curves were used for survival analysis of adverse events, and the difference among the 2 groups (cTnI positive and negative) were compared by log-rank test. Differences were considered statistically significant at the 2-sided *P* < .05 level.

## 3. Results

### 3.1. Patient characteristics

During the study period, a total of 5,951,626 patients visited the EDs, and 7233 patients were diagnosed with SVT. Among them, 128 adult ESKD patients with regular SVT were enrolled, and 62 patients were qualified for inclusion (Fig. 1). Fifty-seven patients (91.9%) were tested for cTnI, and 5 patients were not (Table 1). Patients tested for cTnI were older (*P* = .03) and had a longer length of hospital stay (*P* < .001) compared with those not tested for cTnI. Among those tested for cTnI, 47 patients (82.5%) had a positive result, and 10 (17.5%) had a negative result. Patients’ demographic characteristics are described in Table 2. More comorbidities were present in the cTnI-positive group compared to the cTnI-negative group. Patients with an elevated level of cTnI also had more cardiologist consult (14/47, 24.6%), observation unit stay (20/47, 42.6%), and admission (20/47, 42.6%). Among the cTnI-positive group, only 1 (2.1%) patient died because of dengue fever complicated with septic shock and gastrointestinal bleeding.

**Table 1:** Comparison between cardiac troponin I measured or not in patients with supraventricular tachycardia.

Variable	cTnI measured, n = 57	cTnI not measured, n = 5	*P* value
Age (yr)	65.19 ± 13.96	51.00 ± 13.29	.03*
Male	24 (42.1)	4 (80.0)	.166
Length of stay (h)	107.25 ± 194.18	1.43 ± 0.64	<.001*

Data are presented as mean ± SD or number (percentage).

cTnI = cardiac troponin I.

* *P* < .05.

**Table 2:** Patient characteristics according to cardiac troponin I levels.

Variable	cTnI(+) n = 47 (≥0.04 ng/mL)	cTnI(−) n = 10 (0.04 ng/mL)	*P* value
Age (yr)	65.5 ± 14.9	63.6 ± 7.6	.418
Male	21(44.7)	3(30.0)	.49
Initial heart rate (beats/min)	158.2 ± 30.3	150.4 ± 34.7	.646
Peak heart rate (beats/min)	167.9 ± 17.8	152.2 ± 34.9	.412
Systolic blood pressure (mm Hg)	122.8 ± 33.2	114.0 ± 33.9	.599
Diastolic blood pressure (mm Hg)	82.0 ± 23.5	67.7 ± 16.4	.088
Peritoneal dialysis	5(10.6)	3(30.0)	.149
Length of stay (h)	102.2 ± 186.3	134.0 ± 243.0	.491
Palpitation during hemodialysis	19(40.4)	2(20.0)	.295
RFA in 3 yr	16(34.0)	1(10.0)	.247
SVT duration since ED (min)	57.7 ± 71.3	74.9 ± 84.9	.878
Aspirin or clopidogrel use	16(34.0)	2(20.0)	.704
ST depression*	26(55.3)	4(40.0)	.708
**Initial presentation**			
Palpitation	35(74.5)	8(80.0)	.427
Chest pain	23(48.9)	5(50.0)	.730
Dyspnea	11(23.4)	2(20.0)	1.000
Syncope	1(2.1)	1(10.0)	.293
**Previous medical history**			
Previous SVT	3(6.4)	0(0.0)	1.000
Coronary artery disease	14(29.8)	1(10.0)	.420
Hyperlipidemia	17(36.2)	4(40.0)	.712
Hypertension	43(91.5)	6(60.0)	.103
Diabetes mellitus	19(40.4)	4(40.0)	1.000
Smoking history	8(17.0)	1(10.0)	1.000
**Treatment**			
Spontaneous resolution	5(10.6)	2(20.0)	.304
Adenosine use	40(85.1)	5(50.0)	.082
Verapamil use	6(12.8)	2(20.0)	.599
Amiodarone use	1(23.4)	2(20.0)	1.000
**Disposition**			
Cardiologist consult	14(29.8)	0(0)	.095
Discharge	7(14.9)	4(40.0)	.088
Observation unit	20(42.6)	3(30.0)	.726
Ordinary medical ward	17(36.2)	3(30.0)	1.000
Intensive care unit	3(6.4)	0(0)	1.000
Death	1(2.1)	0	1.000

Data are presented as mean ± SD or number (percentage).

cTnI = cardiac troponin I, ED = emergency department, SVT = supraventricular tachycardia, RFA = radiofrequency ablation.

* ST depression defined as the trace in the ST segment in 2 continuous leads is low below the baseline >1 mm.

Laboratory results revealed no main difference between the 2 groups, except that a calcium level (9.3 mg/dL vs 10.6 mg/dL) in the patients with elevated cTnI level was significantly lower than the cTnI-negative group (*P* = .027) (Table 3). Patients who underwent thyroid function tests were all normal. Only 1 patient with an elevated cTnI level (2.1%) was treated for electrolyte imbalance. Echocardiography was performed in 52 patients (92.1%). Patients with positive cTnI value had significantly lower left ventricular ejection fraction (LVEF) than those with negative value (*P* = .024). In addition, a larger left ventricular end-diastolic diameter was also found in the cTnI-positive group (*P* = .037) (Table 3).

**Table 3:** Analysis of laboratory and echocardiographic finding in patients with or without elevated cardiac troponin I levels.

Variable	cTnI(+) (≥0.04 ng/mL)	cTnI(−) (<0.04 ng/mL)	*P* value
**Laboratory exam**			
White cell count (10^3^/µL) n = 54	9.51 ± 5.79	8.98 ± 5.02	.816
Hemoglobin (g/dL) n = 55	11.5 ± 1.85	11.47 ± 1.89	.937
Platelet (10^3^/µL) n = 54	182.7 ± 75.1	194.2 ± 130.4	.754
Blood urea nitrogen (mg/dL) n = 34	48.30 ± 31.98†	69.88 ± 28.59†	.074
Creatinine (mg/dL) n = 27	7.94 ± 4.42†	9.08 ± 4.92†	.464
Sodium (mEq/L) n = 56	136.3 ± 3.4	135.3 ± 4.3	.711
Potassium (mEq/L) n = 57	4.2 ± 0.7	4.2 ± 0.6	.751
Calcium (mg/dL) n = 25	9.30 ± 0.97	10.60 ± 0.01†	.027
Magnesium (mEq/L) n = 9	1.86 ± 0.35	–	–
AST (U/L) n = 8	59.00 ± 77.00†	35.00 ± 21.21	1.000
ALT (U/L) n = 28	30.71 ± 34.38	27.00 ± 10.42	.355
Albumin (g/dL) n = 7	3.86 ± 0.38	4.11 ± 0.02	.381
Initial cTnI (ng/mL) n = 57	0.274 ± 0.652†	0.026 ± 0.009†	<.001
Peak cTnI n = 57	0.715 ± 1.906†	0.026 ± 0.009†	<.001
TSH (uIU/mL) n = 13	2.26 ± 1.53	–	–
Free thyroxine (ng/dL) n = 12	1.09 ± 0.22	–	–
**Echocardiography**‡			
LVEF (%)	58.18 ± 15.35	72.09 ± 7.52	.024*
LVEF < 50%	12(25.0)	0(0)	.181
LA diameter (mm)	42.14 ± 6.91	38.33 ± 6.71	.154
LVEDD (mm)	50.39 ± 9.36	43.60 ± 3.78	.037*
LVH	20(42.6)	1(10.0)	.219

Data are presented as mean ± SD, number and percentage.

ALT = alanine aminotransferase, AST = aspartate aminotransferase, cTnI = cardiac troponin I, LA = left atrium, LVEDD = left ventricular end-diastolic diameter, LVEF = left ventricular ejection fraction, LVH = left ventricular hypertrophy, TSH = thyroid-stimulating hormone.

* *P* < .05.

† Increased value.

‡ Number = 52 (91.2%).

Among those receiving further investigation within 1 year (21/57, 36.8%), 18 patients (38.3%) had positive cTnI values, and 3 patients (30%) had negative values. No patient of the 2 groups was arranged for a treadmill exercise test. For the patients with elevated levels of cTnI, 14 (29.8%) received the thallium scan, and 10 (21.3%) underwent cardiac catheterization. Only 2 (14.3%) out of 14 thallium scans were positive, and both patients received further cardiac catheterization. The results revealed that 1 had 3-vessel-disease without restenosis of the stent, and the other had 2-vessel-disease with new stent implantation. Of the other 8 patients who directly underwent cardiac catheterization, 2 (25%) had coronary artery disease with stent implantation, and 6 (75%) were patent coronary artery or insignificant stenosis. Of the 3 negative cTnI patients, they all received thallium scans without additional cardiac imaging, and the results were all negative.

### 3.2. Predictors of elevated cTnI level

In univariate analysis, only decreased LVEF (OR = 0.91; CI 0.83–0.99; *P* = .039) was the predictor of elevated cTnI level. In multivariate analysis, we adjusted covariates, including a history of hypertension, peak heart rate, and LVEF, using the stepwise selection method. Independent predictors were a history of hypertension (OR 34.8; CI 2.10–575; *P* = .013) and LVEF (OR 0.86; 0.75–0.99; *P* = .048). A trend toward elevated cTnI level was found in the patients with higher peak heart rate (OR 1.03; CI 0.99–1.07; *P* = .106) (Table 4).

**Table 4:** Multivariate logistic regression results for elevated cardiac troponin I levels.

	Univariate		Multivariate	
	OR (95% CI)	*P* value	OR (95% CI)	*P* value
Age > 65	1.03(0.25,4.31)	.969		
Male	0.64(0.14,2.88)	.563		
Coronary artery disease	3.29(0.38,28.85)	.282		
Hyperlipidemia	0.69(0.16,2.90)	.608		
Hypertension	4.30(0.81,22.8)	.086	34.79(2.10,575.63)	.013*
Diabetes mellitus	0.82(0.20,3.44)	.785		
Smoking history	1.60(0.18,14.63)	.677		
Peak heart rate	1.03(0.99,1.07)	.076	1.03(0.99,1.07)	.106
Palpitation	0.34(0.04,2.96)	.326		
Chest pain	0.74(0.18,3.08)	.675		
ST depression†	2.65(0.56,12.53)	.220		
LVEF (%)	0.91(0.83,0.99)	.039	0.86(0.75,0.99)	.048*

CAD = coronary artery disease, LA = left atrium, LVEF = left ventricular ejection fraction, LVEDD = left ventricular end-diastolic diameter, LVH = left ventricular hypertrophy.

* *P* value < .05.

† ST depression defined as the trace in the ST segment in 2 continuous leads is low below the baseline >1 mm.

### 3.3. Primary endpoint and survival analysis

The mean follow-up period was 20.6 ± 14.7 months. Six of the patients with elevated cTnI levels (12.8%) and none of the patients with negative cTnI values had MACE within 6 weeks (*P* = .575). Among those with MACE in 3 years, 17 patients had positive cTnI value (36.2%), and 2 had negative value (20%), without significant difference (*P* = .703). The composite of MACE in 3 years was described in table 5.

**Table 5:** Adverse outcomes of patients with positive or negative cardiac troponin I.

Variable	cTnI (+) (≥0.04 ng/mL)	cTnI (−) (<0.04 ng/mL)	*P* value
3-yr MACE	17(36.2)	2(20.0)	.703
Ischemic stroke	0(0)	0(0)	–
Revascularization	5(10.6)	0(0)	.582
Acute coronary syndrome	8(17.0)	1(10.0)	1.000
Decompensated heart failure	4(8.5)	0(0)	1.000
Coronary artery bypass graft	0(0)	0(0)	–
Death	12(25.5)	1(10)	.668

Data are presented as number and percentage.

cTnI = cardiac troponin I, MACE = major adverse cardiovascular event.

**Figure 2.**
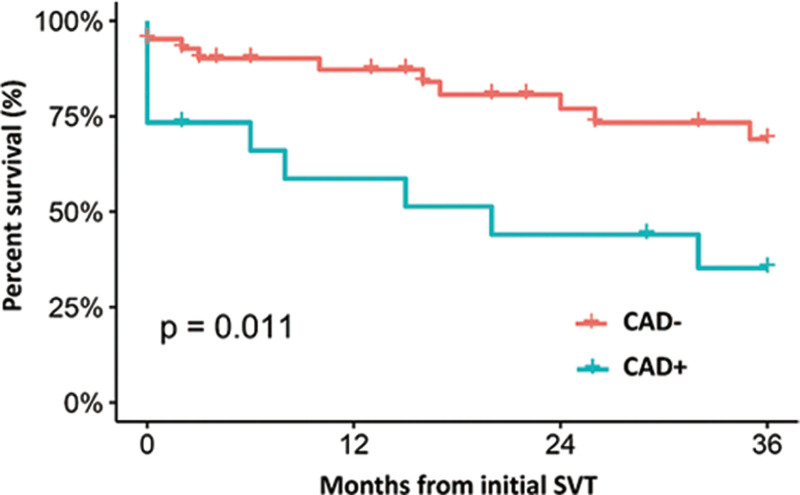
Kaplan–Meier survival curves of patients with or without a history of coronary artery disease for 3-year MACE. MACE = major adverse cardiovascular events.

In Kaplan–Meier analysis, there was no difference in either 6-week MACE (*P* = .297) or 3-year MACE (*P* = .34) between the cTnI-positive and -negative groups. For the primary endpoint of 3-year MACE, univariate Cox regression was performed to identify the possible risk factor. Only the history of coronary artery disease was statistically significant (HR 3.00; CI 1.22–7.40; *P* = .017) (Table 6). Multivariate analysis was not performed, given that no other covariates met the selection criteria. A Kaplan–Meier survival curve of patients with or without a history of coronary artery disease was provided in Figure 2. Subgroup analysis was performed to examine the risk factor for 3-year MACE in patients with elevated cTnI levels. Univariate Cox regression revealed that a history of coronary artery disease (HR 3.34; CI 1.28–8.68; *P* = .014) and diabetes mellitus (HR 2.65; CI 1.01–6.97; *P* = .049) were significant risk factors for 3-year MACE. Multivariate Cox regression revealed that a history of coronary artery disease (HR 2.73; CI 1.01–7.41; *P* = .049) was an independent risk factor for 3-year MACE (Table 7).

**Table 6:** Univariate Cox regression results for 3-year major adverse cardiovascular events.

	Univariate	
	HR (95% CI)	*P* value
Age > 65	0.64 (0.14,2.88)	.563
Male	0.69 (0.27,1.76)	.692
Coronary artery disease	3.00 (1.22,7.40)	.017*
Hyperlipidemia	1.29 (0.52,3.22)	.580
Hypertension	0.55 (0.18,1.68)	.296
Diabetes mellitus	2.03 (0.82,5.00)	.125
Smoking history	0.86 (0.25,2.97)	.814
Peak heart rate	1.01 (0.98,1.02)	.866
Palpitation	0.59 (0.22,1.56)	.289
Chest pain	0.62 (0.24,1.57)	.309
ST depression†	0.46 (0.13,1.63)	.231
LVEF (%)	0.99 (0.96,1.02)	.455

CAD = coronary artery disease, LA = left atrium, LVEDD = left ventricular end-diastolic diameter, LVEF = left ventricular ejection fraction, LVH = left ventricular hypertrophy.

* *P* value < 0.05.

† ST depression defined as the trace in the ST segment in 2 continuous leads is low below the baseline >1 mm.

**Table 7:** Multivariate Cox regression results for 3-year major adverse cardiovascular events in patients with elevated cardiac troponin I levels.

	Univariate		Multivariate	
	HR (95% CI)	*P* value	HR (95% CI)	*P* value
Age > 65	1.62 (0.60,4.38)	.344		
Male	0.75 (0.28,1.97)	.553		
Coronary artery disease	3.34 (1.28,8.68)	.014*	2.73 (1.01,7.41)	.049*
Hyperlipidemia	1.39 (0.53,3.66)	.504		
Hypertension	0.66 (0.15,2.91)	.586		
Diabetes mellitus	2.65 (1.01,6.97)	.049*	2.02 (0.74,5.56)	.172
Smoking history	0.56 (0.13,2.46)	.443		
Peak heart rate	0.99 (0.97,1.02)	.640		
Palpitation	0.55 (0.20,1.49)	.238		
Chest pain	0.57 (0.21,1.54)	.266		
ST depression†	0.10 (0.06,1.28)	.101		
LVEF (%)	0.99 (0.96,1.02)	.639		

CAD = coronary artery disease, LA = left atrium, LVEF = left ventricular ejection fraction, LVEDD = left ventricular end-diastolic diameter, LVH = left ventricular hypertrophy

* *P* value < 0.05.

† ST depression defined as the trace in the ST segment in 2 continuous leads is low below the baseline >1 mm.

## 4. Discussion

To the best of our knowledge, this is the first study to evaluate the prognostic value of troponin in ESKD patients with SVT, as well as the largest report describing the proportion of troponin elevation and detailed clinical presentations in this population. In this study, it observed that many providers performed cTnI testing in ESKD patients presenting with SVT, resulting in increased length of hospital stay, ED crowding, and health care costs compared with those without cTnI testing. Elevation of cTnI in this population is common but may not correlate with adverse outcomes. Among patients with elevated cTnI levels, they have a tendency toward receiving hospitalization and further cardiac catheterization and are at increased risk of antiplatelet or anticoagulant therapy and procedure-related complications without clear benefit. Routine troponin measurement in ESKD patients with SVT is not recommended because it adds little prognostic value to clinical management and risk stratification.

Fernando et al^[10]^ reported a pooled proportion of 32% in the normal population with SVT had positive troponin value in a meta-analysis study. In our study, 82.5% of the dialysis patients had elevated cTnI levels when presenting with SVT. Asymptomatic elevated cTnI level is common in the patients with chronic kidney disease or ESKD.^[18,19]^ About 8% of the dialysis patients was reported to have elevated level of cTnI, and a higher proportion can be seen while using high-sensitive troponin.^[18,19]^ The proportion of elevated troponin level markedly vary in the literature, attributable to different antibodies against troponin epitopes by different manufacturers with various generation immune assays.^[14]^ There are varieties of mechanisms contributing to elevated cTnI levels in dialysis patients. These have been still a source of debate, but generally accepted theories include underlying asymptomatic heart failure, left ventricular hypertrophy, stable coronary artery disease (CAD), uremia-induced myocardial toxicity, and interdialytic volume overload.^[20,21]^ One systemic review and meta-analysis of 98 cohort studies^[22]^ reported that elevated troponin levels in asymptomatic dialysis patients were at increased risk of cardiovascular mortality and all-cause mortality. To address this issue, the U.S. Food and Drug Administration approved monitoring troponin levels to predict prognosis in this population.^[22]^ Nevertheless, this has been a controversial point about routine troponin measurement owing to whether changing clinical management for asymptomatic dialysis patients.

It was widely reported in the literature that elevated cTnI level in SVT patients was not associated higher risk of poor prognosis.^[23]^ Allen et al^[9]^ suggested prudent application of troponin testing in SVT patients due to low cardiovascular risk. However, given the higher prevalence of cardiovascular disease in ESKD patients than the general population,^[24]^ most of the patients with ESKD (91.9%) were tested for cTnI level when presenting SVT in the ED of our institution, resulting in a markedly prolonged length of hospital stay. In the current study, more previous medical histories were present in the cTnI-positive group compared with the cTnI-negative group. There were more cardiologist consultations, shorter observation unit stays, and more hospitalizations in the patients with positive cTnI values.

Most of our physicians performed laboratory investigations of hemoglobin and electrolyte in ESKD patients with SVT in the ED. The association between anemia and SVT has been explored in prior studies, and anemia was recognized as a possible risk factor for SVT.^[11]^ However, only 1 patient had a mild decreased level of hemoglobin, and none of the patients received a blood transfusion in our study. Fluctuating electrolyte levels, including potassium, calcium, and magnesium, in patients receiving hemodialysis was known to cause arrhythmia.^[25]^ In particular, it has been observed that hypokalemia, hyperkalemia, and hypomagnesemia contributed to ventricular arrhythmia as a result of compromised myocardium and effect on the cardiac action potential.^[11,26]^ However, the association between electrolyte abnormalities and SVT has received limited attention in the literature. In our cohort, only 1 patient underwent treatment for mildly elevated potassium levels.

Among patients who underwent the echocardiography examination, cTnI-positive group was associated with significantly reduced LVEF and larger left ventricle end-diastolic diameter compared with cTnI-negative group. Our findings were in line with the previous study conducted by Sharma et al^[27]^ that elevated cTnT level was related to increased left ventricular dimensions and reduced systolic function. The association between impaired systolic function and elevated cTnI level has been largely discussed in the literature.^[28–30]^ The underlying mechanism was believed to be associated with microvascular change and reduced capillary density, contributing to ischemic injury and microinfarct.^[31]^

It is worth noting that elevated cTnI level in ESKD patients could not predict ad-verse outcomes in this study. The only predictor of 3-year MACE was a history of CAD. The relationship between elevated troponin levels in SVT patients with some cardiovascular risk factors and future adverse outcomes remains controversial due to conflicting evidence. Chow et al^[32]^ showed that the rise in cTnI was associated with an increased risk of cardiovascular events in the future. However, the adverse outcomes of the above study were largely attributed to cardiac rehospitalization of some patients presenting with chest pain, atrial fibrillation or flutter, aortic valve re-placement, or pericarditis, and these were not MACEs by definition. Carlberg et al^[23]^ studied 11 patients with positive troponin value in the ED with the diagnosis of SVT and there was no adverse outcome in thirty-day follow-up. Ghersin et al^[13]^ found that positive cTnI value was an independent predictor for adverse outcomes only among patients with a history of CAD.

Our study had several limitations. First, it was retrospective in nature, not allowing us to collect detailed clinical covariates from EMRs though we attempted to mitigate these confounders using multivariate analysis. Second, although there is no agreement about dialysis itself affecting troponin level, we did not compare the status between predialysis and postdialysis in these patients. Third, the study consisted of a relatively small number of patients, and it may limit the overall power to detect a difference in the primary and secondary outcomes and be confounded by several variables, such as patients who did not receive troponin testing. Further studies with a large sample size might be needed to verify the significance of these correlations. Finally, although the acute management of SVT remained the same according to Advanced Cardiac Life Support tachycardia algorithm,^[33]^ the protocol of radiofrequency ablation, cardiac catheterization, CABG, or heart failure management might be different during 12-year study period, which might be potential confounders.^[34]^

## 5. Conclusion

In conclusion, we presented the first study, which identified the prognostic value of troponin elevation in ESKD patients presenting with SVT. A large majority (82.5%) of troponin elevation was observed in this population, but it might have a poor correlation with major adverse cardiovascular events. Patients with positive troponin value frequently underwent further investigations without significant benefit. Physicians should be prudent in ordering troponin testing in these patients unless there is a high index of suspicion for the acute coronary syndrome. Further studies with a large sample size are necessary to strengthen our findings.

## Author contributions

All the authors made substantial contributions for the work. All the authors gave final approval to the submitted paper. All authors agreed to be accountable for all aspects of the work in ensuring that questions related to the accuracy or integrity of the work are appropriately investigated and resolved.

Conceptualization: Chieh-Ching Yen.

**Data curation:** Chih-Kai Wang.

**Formal analysis:** Chieh-Ching Yen.

**Investigation:** Shou-Yen Chen.

**Methodology:** Shou-Yen Chen.

**Project administration:** Hsiang-Yun Lo, Shou-Yen Chen.

**Resources:** Hsiang-Yun Lo.

**Software:** Hsiang-Yun Lo.

**Supervision:** Chip-Jin Ng.

**Validation:** Chip-Jin Ng, Chung-Hsien Chaou.

**Visualization:** Chip-Jin Ng.

**Writing – original draft:** Chih-Kai Wang.

**Writing – review & editing:** Chieh-Ching Yen.
